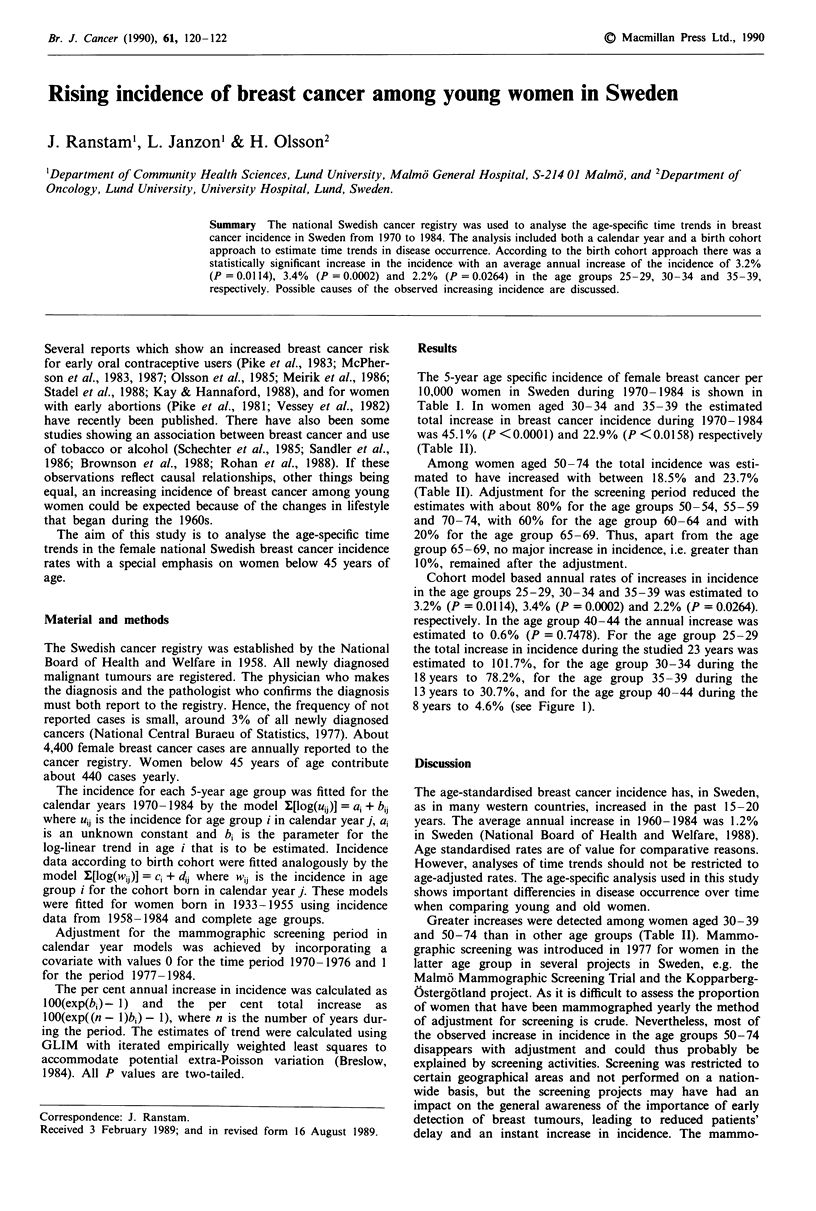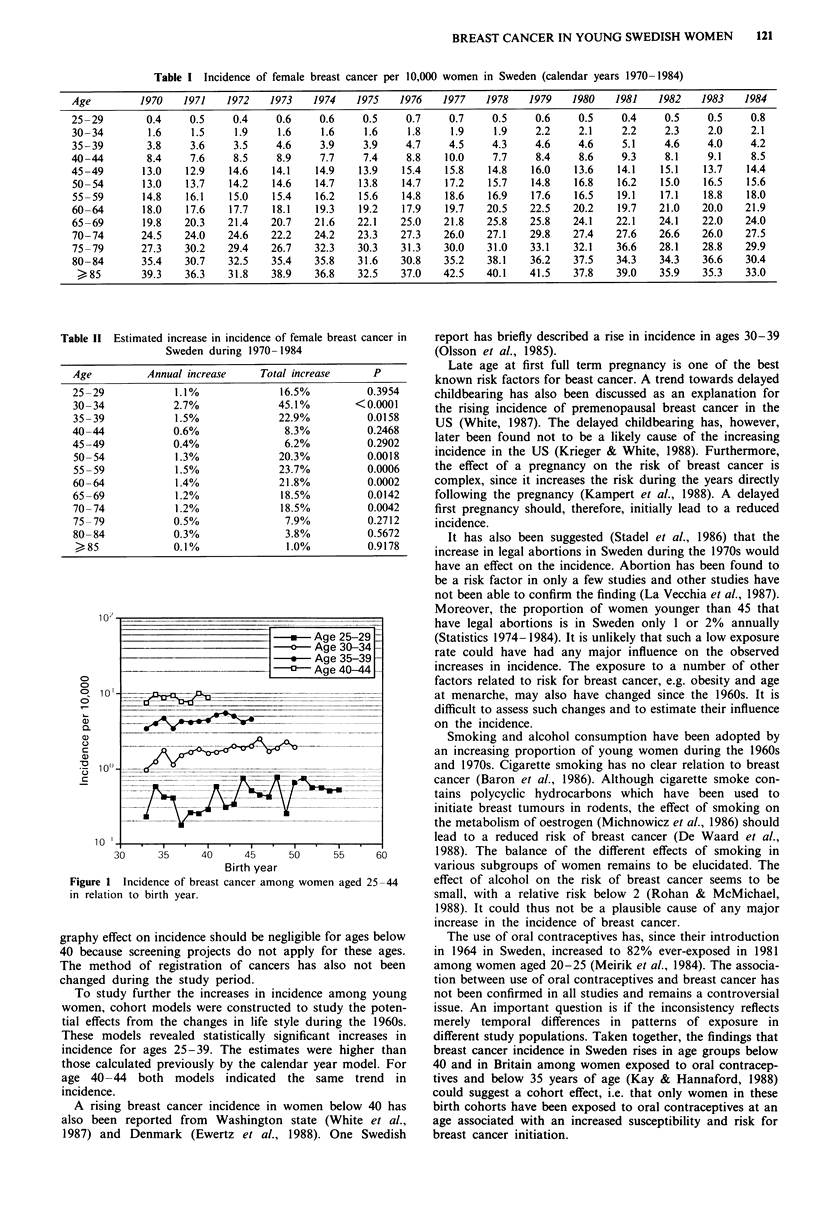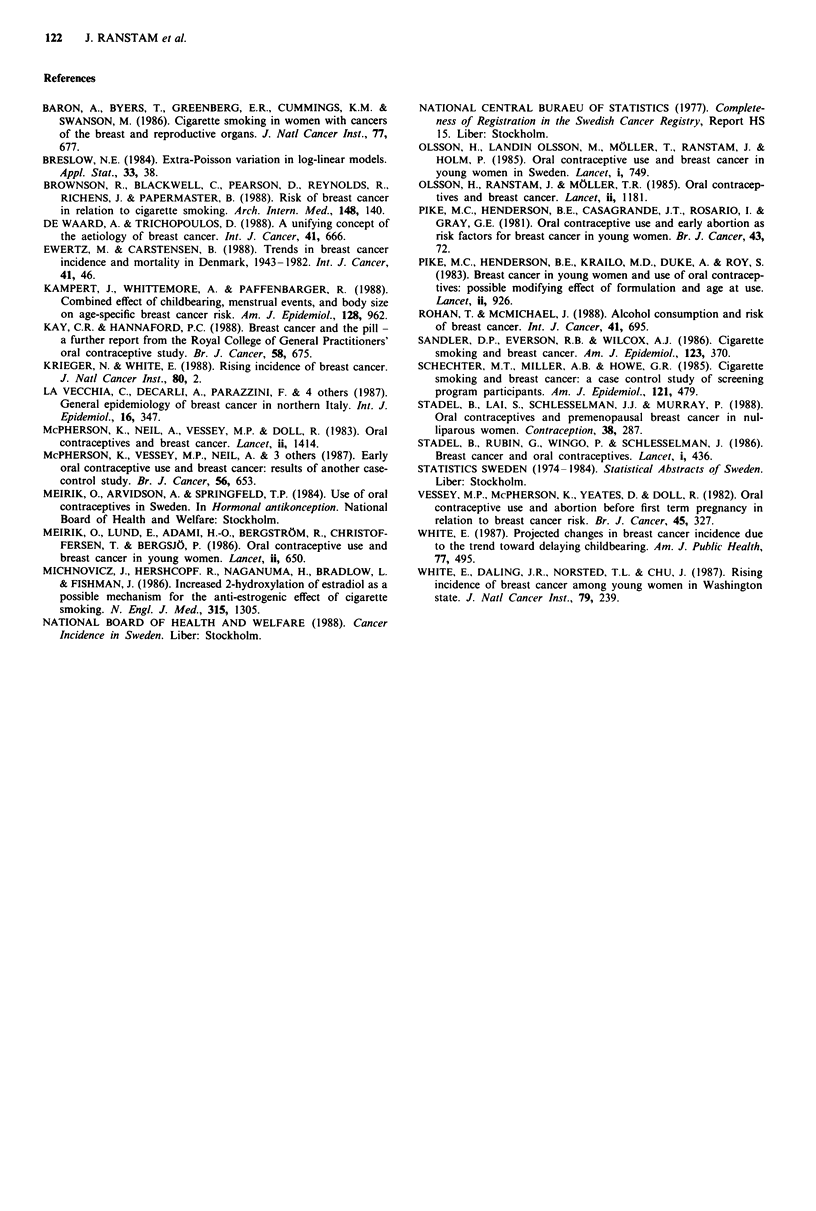# Rising incidence of breast cancer among young women in Sweden.

**DOI:** 10.1038/bjc.1990.24

**Published:** 1990-01

**Authors:** J. Ranstam, L. Janzon, H. Olsson

**Affiliations:** Department of Community Health Sciences, Lund University, Malmö General Hospital, Sweden.

## Abstract

The national Swedish cancer registry was used to analyse the age-specific time trends in breast cancer incidence in Sweden from 1970 to 1984. The analysis included both a calendar year and a birth cohort approach to estimate time trends in disease occurrence. According to the birth cohort approach there was a statistically significant increase in the incidence with an average annual increase of the incidence of 3.2% (P = 0.0114), 3.4% (P = 0.0002) and 2.2% (P = 0.0264) in the age groups 25-29, 30-34 and 35-39, respectively. Possible causes of the observed increasing incidence are discussed.


Br. J. Cancer (1990), 61, 120-122 © Macmillan Press Ltd., 1990

### Rising incidence of breast cancer among young women in Sweden

J. Ranstam[1], L. Janzon[1] & H. Olsson[2]

[1]*Department of Community Health Sciences, Lund University, Malmö General Hospital, S-214 01 Malmö, and* [2]*Department of Oncology, Lund University, University Hospital, Lund, Sweden.*

**Summary** The national Swedish cancer registry was used to analyse the age-specific time trends in breast cancer incidence in Sweden from 1970 to 1984. The analysis included both a calendar year and a birth cohort approach to estimate time trends in disease occurrence. According to the birth cohort approach there was a statistically significant increase in the incidence with an average annual increase of the incidence of 3.2% ($P = 0.0114$), 3.4% ($P = 0.0002$) and 2.2% ($P = 0.0264$) in the age groups 25-29, 30-34 and 35-39, respectively. Possible causes of the observed increasing incidence are discussed.

Several reports which show an increased breast cancer risk for early oral contraceptive users (Pike *et al.*, 1983; McPherson *et al.*, 1983, 1987; Olsson *et al.*, 1985; Meirik *et al.*, 1986; Stadel *et al.*, 1988; Kay & Hannaford, 1988), and for women with early abortions (Pike *et al.*, 1981; Vessey *et al.*, 1982) have recently been published. There have also been some studies showing an association between breast cancer and use of tobacco or alcohol (Schechter *et al.*, 1985; Sandler *et al.*, 1986; Brownson *et al.*, 1988; Rohan *et al.*, 1988). If these observations reflect causal relationships, other things being equal, an increasing incidence of breast cancer among young women could be expected because of the changes in lifestyle that began during the 1960s.

The aim of this study is to analyse the age-specific time trends in the female national Swedish breast cancer incidence rates with a special emphasis on women below 45 years of age.

### Material and methods

The Swedish cancer registry was established by the National Board of Health and Welfare in 1958. All newly diagnosed malignant tumours are registered. The physician who makes the diagnosis and the pathologist who confirms the diagnosis must both report to the registry. Hence, the frequency of not reported cases is small, around 3% of all newly diagnosed cancers (National Central Buraeu of Statistics, 1977). About 4,400 female breast cancer cases are annually reported to the cancer registry. Women below 45 years of age contribute about 440 cases yearly.

The incidence for each 5-year age group was fitted for the calendar years 1970-1984 by the model $\Sigma[\log(u_{ij})] = a_i + b_{ij}$ where $u_{ij}$ is the incidence for age group $i$ in calendar year $j$, $a_i$ is an unknown constant and $b_i$ is the parameter for the log-linear trend in age $i$ that is to be estimated. Incidence data according to birth cohort were fitted analogously by the model $\Sigma[\log(w_{ij})] = c_i + d_{ij}$ where $w_{ij}$ is the incidence in age group $i$ for the cohort born in calendar year $j$. These models were fitted for women born in 1933-1955 using incidence data from 1958-1984 and complete age groups.

Adjustment for the mammographic screening period in calendar year models was achieved by incorporating a covariate with values 0 for the time period 1970-1976 and 1 for the period 1977-1984.

The per cent annual increase in incidence was calculated as $100(\exp(b_i) - 1)$ and the per cent total increase as $100(\exp((n-1)b_i) - 1)$, where $n$ is the number of years during the period. The estimates of trend were calculated using GLIM with iterated empirically weighted least squares to accommodate potential extra-Poisson variation (Breslow, 1984). All $P$ values are two-tailed.

Correspondence: J. Ranstam.
Received 3 February 1989; and in revised form 16 August 1989.

### Results

The 5-year age specific incidence of female breast cancer per 10,000 women in Sweden during 1970-1984 is shown in Table I. In women aged 30-34 and 35-39 the estimated total increase in breast cancer incidence during 1970-1984 was 45.1% ($P < 0.0001$) and 22.9% ($P < 0.0158$) respectively (Table II).

Among women aged 50-74 the total incidence was estimated to have increased with between 18.5% and 23.7% (Table II). Adjustment for the screening period reduced the estimates with about 80% for the age groups 50-54, 55-59 and 70-74, with 60% for the age group 60-64 and with 20% for the age group 65-69. Thus, apart from the age group 65-69, no major increase in incidence, i.e. greater than 10%, remained after the adjustment.

Cohort model based annual rates of increases in incidence in the age groups 25-29, 30-34 and 35-39 was estimated to 3.2% ($P = 0.0114$), 3.4% ($P = 0.0002$) and 2.2% ($P = 0.0264$). respectively. In the age group 40-44 the annual increase was estimated to 0.6% ($P = 0.7478$). For the age group 25-29 the total increase in incidence during the studied 23 years was estimated to 101.7%, for the age group 30-34 during the 18 years to 78.2%, for the age group 35-39 during the 13 years to 30.7%, and for the age group 40-44 during the 8 years to 4.6% (see Figure 1).

### Discussion

The age-standardised breast cancer incidence has, in Sweden, as in many western countries, increased in the past 15-20 years. The average annual increase in 1960-1984 was 1.2% in Sweden (National Board of Health and Welfare, 1988). Age standardised rates are of value for comparative reasons. However, analyses of time trends should not be restricted to age-adjusted rates. The age-specific analysis used in this study shows important differencies in disease occurrence over time when comparing young and old women.

Greater increases were detected among women aged 30-39 and 50-74 than in other age groups (Table II). Mammographic screening was introduced in 1977 for women in the latter age group in several projects in Sweden, e.g. the Malmö Mammographic Screening Trial and the Kopparberg-Östergötland project. As it is difficult to assess the proportion of women that have been mammographed yearly the method of adjustment for screening is crude. Nevertheless, most of the observed increase in incidence in the age groups 50-74 disappears with adjustment and could thus probably be explained by screening activities. Screening was restricted to certain geographical areas and not performed on a nationwide basis, but the screening projects may have had an impact on the general awareness of the importance of early detection of breast tumours, leading to reduced patients' delay and an instant increase in incidence. The mammo-

**Table I** Incidence of female breast cancer per 10,000 women in Sweden (calendar years 1970–1984)

| *Age* | *1970* | *1971* | *1972* | *1973* | *1974* | *1975* | *1976* | *1977* | *1978* | *1979* | *1980* | *1981* | *1982* | *1983* | *1984* |
|---|---|---|---|---|---|---|---|---|---|---|---|---|---|---|---|
| 25–29 | 0.4 | 0.5 | 0.4 | 0.6 | 0.6 | 0.5 | 0.7 | 0.7 | 0.5 | 0.6 | 0.5 | 0.4 | 0.5 | 0.5 | 0.8 |
| 30–34 | 1.6 | 1.5 | 1.9 | 1.6 | 1.6 | 1.6 | 1.8 | 1.9 | 1.9 | 2.2 | 2.1 | 2.2 | 2.3 | 2.0 | 2.1 |
| 35–39 | 3.8 | 3.6 | 3.5 | 4.6 | 3.9 | 3.9 | 4.7 | 4.5 | 4.3 | 4.6 | 4.6 | 5.1 | 4.6 | 4.0 | 4.2 |
| 40–44 | 8.4 | 7.6 | 8.5 | 8.9 | 7.7 | 7.4 | 8.8 | 10.0 | 7.7 | 8.4 | 8.6 | 9.3 | 8.1 | 9.1 | 8.5 |
| 45–49 | 13.0 | 12.9 | 14.6 | 14.1 | 14.9 | 13.9 | 15.4 | 15.8 | 14.8 | 16.0 | 13.6 | 14.1 | 15.1 | 13.7 | 14.4 |
| 50–54 | 13.0 | 13.7 | 14.2 | 14.6 | 14.7 | 13.8 | 14.7 | 17.2 | 15.7 | 14.8 | 16.8 | 16.2 | 15.0 | 16.5 | 15.6 |
| 55–59 | 14.8 | 16.1 | 15.0 | 15.4 | 16.2 | 15.6 | 14.8 | 18.6 | 16.9 | 17.6 | 16.5 | 19.1 | 17.1 | 18.8 | 18.0 |
| 60–64 | 18.0 | 17.6 | 17.7 | 18.1 | 19.3 | 19.2 | 17.9 | 19.7 | 20.5 | 22.5 | 20.2 | 19.7 | 21.0 | 20.0 | 21.9 |
| 65–69 | 19.8 | 20.3 | 21.4 | 20.7 | 21.6 | 22.1 | 25.0 | 21.8 | 25.8 | 25.8 | 24.1 | 22.1 | 24.1 | 22.0 | 24.0 |
| 70–74 | 24.5 | 24.0 | 24.6 | 22.2 | 24.2 | 23.3 | 27.3 | 26.0 | 27.1 | 29.8 | 27.4 | 27.6 | 26.6 | 26.0 | 27.5 |
| 75–79 | 27.3 | 30.2 | 29.4 | 26.7 | 32.3 | 30.3 | 31.3 | 30.0 | 31.0 | 33.1 | 32.1 | 36.6 | 28.1 | 28.8 | 29.9 |
| 80–84 | 35.4 | 30.7 | 32.5 | 35.4 | 35.8 | 31.6 | 30.8 | 35.2 | 38.1 | 36.2 | 37.5 | 34.3 | 34.3 | 36.6 | 30.4 |
| ⩾85 | 39.3 | 36.3 | 31.8 | 38.9 | 36.8 | 32.5 | 37.0 | 42.5 | 40.1 | 41.5 | 37.8 | 39.0 | 35.9 | 35.3 | 33.0 |

**Table II** Estimated increase in incidence of female breast cancer in Sweden during 1970–1984

| *Age* | *Annual increase* | *Total increase* | *P* |
|---|---|---|---|
| 25–29 | 1.1% | 16.5% | 0.3954 |
| 30–34 | 2.7% | 45.1% | <0.0001 |
| 35–39 | 1.5% | 22.9% | 0.0158 |
| 40–44 | 0.6% | 8.3% | 0.2468 |
| 45–49 | 0.4% | 6.2% | 0.2902 |
| 50–54 | 1.3% | 20.3% | 0.0018 |
| 55–59 | 1.5% | 23.7% | 0.0006 |
| 60–64 | 1.4% | 21.8% | 0.0002 |
| 65–69 | 1.2% | 18.5% | 0.0142 |
| 70–74 | 1.2% | 18.5% | 0.0042 |
| 75–79 | 0.5% | 7.9% | 0.2712 |
| 80–84 | 0.3% | 3.8% | 0.5672 |
| ⩾85 | 0.1% | 1.0% | 0.9178 |

**Figure 1** Incidence of breast cancer among women aged 25–44 in relation to birth year.

graphy effect on incidence should be negligible for ages below 40 because screening projects do not apply for these ages. The method of registration of cancers has also not been changed during the study period.

To study further the increases in incidence among young women, cohort models were constructed to study the potential effects from the changes in life style during the 1960s. These models revealed statistically significant increases in incidence for ages 25–39. The estimates were higher than those calculated previously by the calendar year model. For age 40–44 both models indicated the same trend in incidence.

A rising breast cancer incidence in women below 40 has also been reported from Washington state (White *et al.*, 1987) and Denmark (Ewertz *et al.*, 1988). One Swedish report has briefly described a rise in incidence in ages 30–39 (Olsson *et al.*, 1985).

Late age at first full term pregnancy is one of the best known risk factors for beast cancer. A trend towards delayed childbearing has also been discussed as an explanation for the rising incidence of premenopausal breast cancer in the US (White, 1987). The delayed childbearing has, however, later been found not to be a likely cause of the increasing incidence in the US (Krieger & White, 1988). Furthermore, the effect of a pregnancy on the risk of breast cancer is complex, since it increases the risk during the years directly following the pregnancy (Kampert *et al.*, 1988). A delayed first pregnancy should, therefore, initially lead to a reduced incidence.

It has also been suggested (Stadel *et al.*, 1986) that the increase in legal abortions in Sweden during the 1970s would have an effect on the incidence. Abortion has been found to be a risk factor in only a few studies and other studies have not been able to confirm the finding (La Vecchia *et al.*, 1987). Moreover, the proportion of women younger than 45 that have legal abortions is in Sweden only 1 or 2% annually (Statistics 1974–1984). It is unlikely that such a low exposure rate could have had any major influence on the observed increases in incidence. The exposure to a number of other factors related to risk for breast cancer, e.g. obesity and age at menarche, may also have changed since the 1960s. It is difficult to assess such changes and to estimate their influence on the incidence.

Smoking and alcohol consumption have been adopted by an increasing proportion of young women during the 1960s and 1970s. Cigarette smoking has no clear relation to breast cancer (Baron *et al.*, 1986). Although cigarette smoke contains polycyclic hydrocarbons which have been used to initiate breast tumours in rodents, the effect of smoking on the metabolism of oestrogen (Michnowicz *et al.*, 1986) should lead to a reduced risk of breast cancer (De Waard *et al.*, 1988). The balance of the different effects of smoking in various subgroups of women remains to be elucidated. The effect of alcohol on the risk of breast cancer seems to be small, with a relative risk below 2 (Rohan & McMichael, 1988). It could thus not be a plausible cause of any major increase in the incidence of breast cancer.

The use of oral contraceptives has, since their introduction in 1964 in Sweden, increased to 82% ever-exposed in 1981 among women aged 20–25 (Meirik *et al.*, 1984). The association between use of oral contraceptives and breast cancer has not been confirmed in all studies and remains a controversial issue. An important question is if the inconsistency reflects merely temporal differences in patterns of exposure in different study populations. Taken together, the findings that breast cancer incidence in Sweden rises in age groups below 40 and in Britain among women exposed to oral contraceptives and below 35 years of age (Kay & Hannaford, 1988) could suggest a cohort effect, i.e. that only women in these birth cohorts have been exposed to oral contraceptives at an age associated with an increased susceptibility and risk for breast cancer initiation.

### References

BARON, A., BYERS, T., GREENBERG, E.R., CUMMINGS, K.M. & SWANSON, M. (1986). Cigarette smoking in women with cancers of the breast and reproductive organs. *J. Natl Cancer Inst.*, **77**, 677.

BRESLOW, N.E. (1984). Extra-Poisson variation in log-linear models. *Appl. Stat.*, **33**, 38.

BROWNSON, R., BLACKWELL, C., PEARSON, D., REYNOLDS, R., RICHENS, J. & PAPERMASTER, B. (1988). Risk of breast cancer in relation to cigarette smoking. *Arch. Intern. Med.*, **148**, 140.

DE WAARD, A. & TRICHOPOULOS, D. (1988). A unifying concept of the aetiology of breast cancer. *Int. J. Cancer*, **41**, 666.

EWERTZ, M. & CARSTENSEN, B. (1988). Trends in breast cancer incidence and mortality in Denmark, 1943–1982. *Int. J. Cancer*, **41**, 46.

KAMPERT, J., WHITTEMORE, A. & PAFFENBARGER, R. (1988). Combined effect of childbearing, menstrual events, and body size on age-specific breast cancer risk. *Am. J. Epidemiol.*, **128**, 962.

KAY, C.R. & HANNAFORD, P.C. (1988). Breast cancer and the pill – a further report from the Royal College of General Practitioners' oral contraceptive study. *Br. J. Cancer*, **58**, 675.

KRIEGER, N. & WHITE, E. (1988). Rising incidence of breast cancer. *J. Natl Cancer Inst.*, **80**, 2.

LA VECCHIA, C., DECARLI, A., PARAZZINI, F. & 4 others (1987). General epidemiology of breast cancer in northern Italy. *Int. J. Epidemiol.*, **16**, 347.

McPHERSON, K., NEIL, A., VESSEY, M.P. & DOLL, R. (1983). Oral contraceptives and breast cancer. *Lancet*, **ii**, 1414.

McPHERSON, K., VESSEY, M.P., NEIL, A. & 3 others (1987). Early oral contraceptive use and breast cancer: results of another case-control study. *Br. J. Cancer*, **56**, 653.

MEIRIK, O., ARVIDSON, A. & SPRINGFELD, T.P. (1984). Use of oral contraceptives in Sweden. In *Hormonal antikonception*. National Board of Health and Welfare: Stockholm.

MEIRIK, O., LUND, E., ADAMI, H.-O., BERGSTRÖM, R., CHRISTOFFERSEN, T. & BERGSJÖ, P. (1986). Oral contraceptive use and breast cancer in young women. *Lancet*, **ii**, 650.

MICHNOVICZ, J., HERSHCOPF. R., NAGANUMA, H., BRADLOW, L. & FISHMAN, J. (1986). Increased 2-hydroxylation of estradiol as a possible mechanism for the anti-estrogenic effect of cigarette smoking. *N. Engl. J. Med.*, **315**, 1305.

NATIONAL BOARD OF HEALTH AND WELFARE (1988). *Cancer Incidence in Sweden*. Liber: Stockholm.

NATIONAL CENTRAL BURAEU OF STATISTICS (1977). *Completeness of Registration in the Swedish Cancer Registry*, Report HS 15. Liber: Stockholm.

OLSSON, H., LANDIN OLSSON, M., MÖLLER, T., RANSTAM, J. & HOLM, P. (1985). Oral contraceptive use and breast cancer in young women in Sweden. *Lancet*, **i**, 749.

OLSSON, H., RANSTAM, J. & MÖLLER, T.R. (1985). Oral contraceptives and breast cancer. *Lancet*, **ii**, 1181.

PIKE, M.C., HENDERSON, B.E., CASAGRANDE, J.T., ROSARIO, I. & GRAY, G.E. (1981). Oral contraceptive use and early abortion as risk factors for breast cancer in young women. *Br. J. Cancer*, **43**, 72.

PIKE, M.C., HENDERSON, B.E., KRAILO, M.D., DUKE, A. & ROY, S. (1983). Breast cancer in young women and use of oral contraceptives: possible modifying effect of formulation and age at use. *Lancet*, **ii**, 926.

ROHAN, T. & MCMICHAEL, J. (1988). Alcohol consumption and risk of breast cancer. *Int. J. Cancer*, **41**, 695.

SANDLER, D.P., EVERSON, R.B. & WILCOX, A.J. (1986). Cigarette smoking and breast cancer. *Am. J. Epidemiol.*, **123**, 370.

SCHECHTER, M.T., MILLER, A.B. & HOWE, G.R. (1985). Cigarette smoking and breast cancer: a case control study of screening program participants. *Am. J. Epidemiol.*, **121**, 479.

STADEL, B., LAI, S., SCHLESSELMAN, J.J. & MURRAY, P. (1988). Oral contraceptives and premenopausal breast cancer in nulliparous women. *Contraception*, **38**, 287.

STADEL, B., RUBIN, G., WINGO, P. & SCHLESSELMAN, J. (1986). Breast cancer and oral contraceptives. *Lancet*, **i**, 436.

STATISTICS SWEDEN (1974–1984). *Statistical Abstracts of Sweden*. Liber: Stockholm.

VESSEY, M.P., McPHERSON, K., YEATES, D. & DOLL, R. (1982). Oral contraceptive use and abortion before first term pregnancy in relation to breast cancer risk. *Br. J. Cancer*, **45**, 327.

WHITE, E. (1987). Projected changes in breast cancer incidence due to the trend toward delaying childbearing. *Am. J. Public Health*, **77**, 495.

WHITE, E., DALING, J.R., NORSTED, T.L. & CHU, J. (1987). Rising incidence of breast cancer among young women in Washington state. *J. Natl Cancer Inst.*, **79**, 239.